# Coexistence of diabetes mellitus and pre-existing cardiovascular disease and mortality in Chinese patients on peritoneal dialysis

**DOI:** 10.1186/s12882-022-02702-0

**Published:** 2022-02-17

**Authors:** Guangtao Lei, Xiaoran Feng, Xiaoyang Wang, Yueqiang Wen, FenFen Peng, Niansong Wang, Xiaojiang Zhan, Qinghua Wu, Xianfeng Wu

**Affiliations:** 1 Department of Cardiology, The Second Affiliated Hospital of Nanchang University, Nanchang, China; 2 Department of Nephrology, Jiujiang No. 1 People’s Hospital, Jiujiang, China; 3 Department of Nephrology, The First Affiliated Hospital of Zhengzhou University, Zhengzhou, China; 4 Department of Nephrology, The Second Affiliated Hospital of Guangzhou Medical University, Guangzhou, China; 5 Department of Nephrology, Zhujiang Hospital of Southern Medical University, Guangzhou, China; 6 Department of Nephrology, Affiliated Sixth People’s Hospital, Shanghai Jiao Tong University, Shanghai, China; 7 Department of Nephrology, the First Affiliated Hospital of Nanchang University, Nanchang, China

**Keywords:** Cardiovascular disease, Diabetes mellitus, Mortality, Peritoneal dialysis

## Abstract

**Background:**

Little is known about the association between the coexistence of diabetes mellitus (DM) and pre-existing cardiovascular disease (CVD) and mortality in patients undergoing continuous ambulatory peritoneal dialysis (CAPD).

**Methods:**

A retrospective cohort study of 2939 Chinese incident CAPD patients was conducted between January 1, 2005, and December 31, 2018. The primary and secondary outcomes were all-cause and CVD mortality. The association between the coexistence of DM and pre-existing CVD and mortality was evaluated using Cox proportional hazards regression.

**Results:**

Over a median of 35.1 months of follow-up, 519 patients (17.7%) died, with 258 (8.8%) being CVD-related deaths. DM plus pre-existing CVD, DM, and pre-existing CVD were associated with a higher risk of all-cause mortality (adjusted hazard ratio [HR], 2.85; 95% confidence interval [CI], 2.18 to 3.72; adjusted HR, 1.89; 95% CI, 1.50 to 2.38; and HR, 1.43; 95% CI, 1.07 to 1.92; P for tend < 0.001) and CVD mortality (adjusted HR, 2.79; 95% CI, 1.91 to 4.08; HR, 1.88; 95% CI, 1.35 to 2.61; and HR, 1.82; 95% CI, 1.23 to 2.68; P for trend < 0.001) than no DM or pre-existing CVD. Subgroup analyses stratified by sex, hypertension status, and hyperlipidemia status showed a similar pattern.

**Conclusions:**

The coexistence of DM and pre-existing CVD at the start of CAPD was more strongly associated with a higher risk of all-cause and CVD mortality than DM or pre-existing CVD alone.

## Background

Although renal replacement treatment improved significantly in recent decades, dialysis patients' overall prognosis remains poor, with 40% of deaths being due to cardiovascular disease (CVD) in this population [1]. Dialysis patients have 10 to 30 times higher CVD mortality than the general population [2]. Thus, managing CVD risks is a significant part of caring for dialysis patients. Many CVD risk factors (such as diabetes mellitus [DM], hypertension, pre-existing CVD, dyslipidemia, and obesity) have been found to be more common among individuals undergoing dialysis than in the general population.

DM and pre-existing CVD are independently associated with the mortality of the dialysis population [3–6]. A recent meta-analysis with 23 studies assessing 86,915 dialysis patients showed that compared with non-DM dialysis patients, DM dialysis patients have 2.00-fold all-cause mortality and 2.11-fold CVD mortality, and those with pre-existing CVD have 1.41-fold all-cause mortality and 2.53-fold CVD mortality compared with those without pre-existing CVD [7]. Previous studies also reported that pre-existing CVD was a risk factor for mortality in general DM patients and dialysis patients [8, 9]. However, the association between the coexistence of DM and pre-existing CVD and mortality has received little attention in peritoneal dialysis patients. We hypothesized that the coexistence of DM and pre-existing CVD was more strongly associated with mortality than DM or pre-existing CVD alone. Therefore, we aimed to examine the effect of the coexistence of DM and pre-existing CVD on mortality at the start of dialysis in patients undergoing continuous ambulatory peritoneal dialysis (CAPD).

## Materials and methods

### Study design and population

A retrospective cohort study with 3073 Chinese incident CAPD patients from five peritoneal dialysis centers in three provinces in China was conducted between January 1, 2005, and December 31, 2018. To increase the generalizability of the CAPD population's findings, we excluded only those aged < 18 years or with less than three months of follow-up. The Human Ethics Committee approved each research facility's study, consistent with the Declaration of Helsinki's ethical principles.

### Data collection and follow Up

Data at the start of CAPD were extracted from medical records by two trained investigators in each facility using uniform and standardized data collection tools. Data included demographic characteristics (age, sex, body mass index, systolic blood pressure [BP], diastolic BP, 24-h urine volume, current smoking, and current alcohol consumption); comorbidities (diabetes mellitus [DM], pre-existing CVD, hypertension, hyperlipidemia); medications (calcium channel blockers, beta-blockers, angiotensin II receptor blockers/angiotensin-converting enzyme inhibitors [ACEI/ARBs], diuretics, statins, and aspirin); and laboratory parameters (hemoglobin, serum albumin, serum uric acid, estimated glomerular filtration rate [eGFR], cholesterol, triglyceride, high-density lipoprotein, low-density lipoprotein, high-sensitivity C-reactive protein [hs-CRP]). All laboratory parameters of fasting blood samples were measured in the departments of each tertiary hospital laboratory.

Patients needed to return to each center at least quarterly for an overall medical assessment, and trained nurses conducted monthly face-to-face interviews or monthly telephone interviews to assess the patient’s overall condition and related medications. All patients were followed up until CAPD cessation, death, eight years of follow-up, or June 30, 2019. Transferring to hemodialysis, receiving renal transplantation, transferring to other centers, loss of follow-up, and survival with an eight-year follow-up prior to June 30, 2019, were considered exclusion criteria.

### Outcomes and definitions

The primary and secondary outcomes were all-cause and CVD mortality, respectively. If the patients died in the hospital, the exact cause of death was identified by death certificates. If the patients died outside a hospital, experts reached a consensus on the cause of death based on the integration of recent health conditions provided by family members and the medical history and descriptions from each dialysis facility.

DM was defined as (1) HbA1c ≥ 6.5%, (2) fasting plasma glucose ≥ 126 mg/dL, (3) 2-h plasma glucose ≥ 200 mg/dL during an OGTT, or (4) in a patient with classic symptoms of hyperglycemia or hyperglycemic crisis, a random plasma glucose ≥ 200 mg/dL when patients met one criterion. In the absence of unequivocal hyperglycemia, criteria 1 to 3 should be confirmed by repeat testing [10]. CVD was defined as coronary heart disease, congestive heart failure, arrhythmias, cerebrovascular disease, or peripheral vascular disease [11]. Hypertension was defined as systolic blood pressure > 140 mmHg, diastolic blood pressure > 90 mmHg, or the use of antihypertensive medications [12]. Hyperlipidemia was defined as serum cholesterol levels ≥ 240 mg/dL, triglyceride levels ≥ 200 mg/dL, low-density lipoprotein levels ≥ 160 mg/dL, or when the patients were receiving lipid-lowering drugs when they met one criterion [13]. Patients with pre-existing CVD using statins were not considered to have hyperlipidemia. Current smoking was defined as at least one cigarette a day, and current alcohol consumption was defined as > 20 g of ethanol a day [14]. eGFR was calculated using the Chronic Kidney Disease Epidemiology Collaboration Eq [15].

### Statistical analysis

The incidence rate was calculated as a proportion of events divided by the total valid observational time at risk, scaled to episodes per 1000 years. Data are presented as the mean ± standard deviation or median (interquartile range, IQR) or number (%). All patients were divided into four groups: the control group (without DM and pre-existing CVD), CVD group (with only pre-existing CVD), DM group (with only DM), and DM plus pre-existing CVD group (with the coexistence of DM and pre-existing CVD). Baseline variables were compared by one-way ANOVA or Kruskal–Wallis tests according to variable distribution (normality tested with Shapiro–Wilk test) for quantitative variables and the chi-square test when appropriate for categorical variables. Multinomial logistic regression was conducted to estimate adjusted odds ratios for the coexistence of DM and pre-existing CVD, DM, and pre-existing CVD versus the control. The following factors were included in multinomial logistic regression: age, sex, body mass index, systolic BP, diastolic BP, current smoking, current alcohol consumption, 24-h urine volume, hypertension, hyperlipidemia, hemoglobin, serum albumin, serum uric acid, eGFR, cholesterol, triglyceride, high-density lipoprotein, low-density lipoprotein, and hs-CRP.

Kaplan–Meier curves were used to examine the difference in survival probability among the four groups over the observational period. Four Cox proportional hazard regression models were conducted to examine the association between the coexistence of DM and pre-existing CVD: Model 1, unadjusted; Model 2, Model 1 plus demographic characteristics, hypertension, and hyperlipidemia; Model 3, Model 2 plus medications; and Model 4, Model 3 plus laboratory parameters. *P* values for trend were examined by treating the four groups as a continuous variable in each model. To evaluate subgroup modification effects on the relationship between coexisting DM and CVD and mortality, we conducted subgroup analyses of the association between the coexistence of DM and pre-existing CVD and mortality stratified by sex, hypertension, nonhypertension, hyperlipidemia, and nonhyperlipidemia. The interaction between the coexistence of DM and CVD and subgroups on all-cause and CVD mortality was examined by conducting a formal interaction test. The Cox proportional hazards results are presented as the hazard ratio (HR) and the 95% confidence interval (CI). The level of significance was set as 0.05 for all analyses. Stata 15.1. statistical software (StataCorp, College Station, TX) was used for the analyses.

## Results

### Baseline characteristics

Of the 3073 potential patients, 42 patients < 18 years and 92 patients with less than three months of follow-up were excluded. The remaining 2939 patients were eligible for the present analysis.

Of 2939 patients with a median age of 50.0 (IQR 39.0–61.0), 1697 (57.7%) were men, 549 (18.7%) had DM, 410 (14.0%) had pre-existing CVD, 1915 (65.2%) had hypertension, and 533 (18.1%) had hyperlipidemia. Eligible patients were assigned to four groups: the DM plus CVD group (*n* = 177, 6.0%), DM group (*n* = 372, 12.7%), CVD group (*n* = 233, 7.9%), and control group (*n* = 2157, 73.4%, Table 1).

**Table 1:** Baseline demographic characteristics, medications, and laboratory parameters

Groups	Study population	Control group	CVD group	DM group	DM plus pre-existing CVD group	*P*-value
N	2939	2157	233	372	177	
Age at study entry, years	50.0 (39.0–61.0)	46.0 (36.0–56.0)	58.0 (45.0–69.0)	59.0 (51.0–66.0)	63.0 (57.0–69.0)	< 0.001
Men, %	1697 (57.7%)	1230 (57.0%)	137 (58.8%)	227 (61.0%)	103 (58.2%)	0.529
Body mass index, kg/m^2^	22.4 ± 3.6	22.1 ± 3.5	21.9 ± 4.0	23.2 ± 3.7	24.5 ± 3.3	< 0.001
Systolic BP, mmHg	150.0 ± 25.6	148.8 ± 25.6	151.6 ± 25.9	153.2 ± 24.5	156.6 ± 25.80	< 0.001
Diastolic BP, mmHg	87.5 ± 15.7	88.8 ± 16.0	87.3 ± 15.7	83.1 ± 13.3	80.8 ± 13.9	< 0.001
24-h urine volume, ml	800(500–1200)	800(500–1200)	800(400–1300)	800 (500–1200)	800 (500–1150)	0.704
Current smoking, (%)	294 (10.00%)	211 (9.78%)	26 (11.16%)	36 (9.68%)	21 (11.86%)	0.756
Current alcohol consumption, (%)	108 (3.7%)	84 (3.9%)	8 (3.4%)	11 (3.0%)	5 (2.8%)	0.745
Hypertension, (%)	1915 (65.2%)	1253 (58.1%)	183 (78.5%)	308 (82.8%)	171 (96.6%)	< 0.001
Hyperlipidemia, (%)	533 (18.1%)	291 (13.5%)	64 (27.5%)	106 (28.5%)	72 (40.7%)	< 0.001
Calcium channel blockers, (%)	2201 (74.9%)	1567 (72.7%)	178 (76.4%)	294 (79.0%)	162 (91.5%)	< 0.001
Beta blockers, (%)	1213 (41.3%)	877 (40.7%)	100 (42.9%)	145 (39.0%)	91 (51.4%)	0.003
Diuretics, (%)	200 (6.8%)	104 (4.8%)	15 (6.4%)	47 (12.6%)	34 (19.2%)	< 0.001
ACEI/ARBs, (%)	1012 (34.4%)	671 (31.1%)	89 (38.2%)	155 (41.7%)	97 (54.8%)	< 0.001
Aspirin, (%)	244 (8.3%)	102 (4.7%)	26 (11.2%)	54 (14.5%)	62 (35.0%)	< 0.001
Statins, (%)	416 (14.2%)	218 (10.1%)	55 (23.6%)	80 (21.5%)	63 (35.6%)	< 0.001
Hemoglobin, g/dL	9.25 ± 2.83	9.11 ± 2.82	9.47 ± 2.80	9.46 ± 2.83	10.16 ± 2.76	< 0.001
Serum albumin, g/dL	3.47 ± 0.56	3.47 ± 0.56	3.47 ± 0.59	3.45 ± 0.56	3.48 ± 0.61	0.939
Serum uric acid, mg/dL	6.92 ± 2.34	6.99 ± 2.37	6.73 ± 2.10	6.70 ± 2.31	6.71 ± 2.32	0.049
eGFR, mL/min/1.73 m^2^	6.44 (4.74–8.34)	6.40 (4.72–8.28)	5.91 (4.43–7.98)	6.71 (4.85–8.60)	6.63 (4.98–8.75)	0.683
Cholesterol, mg/dL	151.6 (117.9–183.0)	148.9 (116.1–181.8)	157.4 (125.3–185.6)	153.2 (119.9–184.5)	157.2 (129.5–187.9)	0.031
Triglyceride, mg/dL	93.9 (55.8–153.3)	93.6 (55.8–151.6)	92.4 (47.8–158.6)	93.3 (56.9–155.5)	114.3 (64.0–158.6)	0.030
High-density lipoprotein, mg/dL	39.6 (31.3–49.5)	39.4 (31.5–49.5)	41.4 (31.8–48.7)	40.5 (30.8–51.8)	37.1 (29.8–47.9)	0.291
Low-density lipoprotein, mg/dL	81.6 (48.0–116.8)	81.3 (49.9–116.1)	79.3 (25.4–116.1)	85.7 (49.0–121.6)	79.3 (29.5–122.2)	0.444
hs-CRP, mg/L	4.37 (1.91–14.18)	4.24 (1.90–13.10)	4.74 (1.93–18.70)	5.00 (2.14–18.75)	4.74 (1.70–18.91)	0.181

*DM* diabetes mellitus, *CVD* cardiovascular disease, *BP* blood pressure, *ACEI/ARB* beta blockers, angiotensin II receptor blockers/angiotensin-converting enzyme inhibitors, *eGFR* estimated glomerular filtration rate, *hs-CRP* high-sensitivity C-reactive protein

Compared to the control group, the DM plus CVD group tended to be older aged; have a higher body mass index, systolic BP, hemoglobin, cholesterol, and triglyceride; have a lower diastolic BP and serum uric acid; and be more likely to have hypertension and hyperlipidemia and use calcium channel blockers, beta-blockers, diuretics, ACEI/ARBs, aspirin, and statins.

### Association of baseline variables and the coexistence of DM and pre-existing CVD

We analyzed the variables associated with the coexistence of DM and pre-existing CVD, DM, and pre-existing CVD versus the control at baseline using multinomial logistic regression (Table 2).

**Table 2:** Associations between baseline variables and the co-existence of HD and pre-existing CVD using the multinomial logistic regression

	Control group	Pre-existing CVD group		DM group		DM plus pre-existing CVD group	
		OR	95% CI	OR	95% CI	OR	95% CI
Age, per increase 10 year	1.0 (ref.)	1.53	1.42 to 1.64	1.52	1.43 to 1.62	1.85	1.70 to 2.10
Women, men as a reference	1.0 (ref.)	0.79	0.58 to 1.06	0.74	0.57 to 0.95	0.78	0.53 to 1.13
BMI, per increase 1 kg/m^2^	1.0 (ref.)	0.96	0.92 to 0.99	1.06	1.03 to 1.10	1.18	1.13 to 1.23
Systolic BP, per increase 10 mmHg	1.0 (ref.)	1.02	0.95 to 1.09	1.12	1.06 to 1.18	1.21	1.12 to 1.31
Diastolic BP, per increase 10 mmHg	1.0 (ref.)	1.01	0.91 to 1.12	0.76	0.69 to 0.83	0.66	0.57 to 0.75
Hypertension, yes/no	1.0 (ref.)	2.26	1.60 to 3.17	2.84	2.10 to 3.84	13.72	6.14 to 30.63
Hyperlipidemia, yes/no	1.0 (ref.)	2.20	1.58 to 3.06	2.31	1.75 to 3.07	3.51	2.41 to 5.12
Hemoglobin, per increase 1 mg/dL	1.0 (ref.)	1.02	0.97 to 1.07	1.02	0.98 to 1.06	1.09	1.03 to 1.16
Serum uric acid, per increase 1 mg/dL	1.0 (ref.)	0.95	0.90 to 1.01	0.94	0.89 to 0.99	0.92	0.86 to 1.00

The following variables at baseline were in the multinomial logistic regression model: age, sex, body mass index, systolic BP, diastolic BP, current smoking, current alcohol consumption, 24-h urine volume, hypertension, hyperlipidemia, hemoglobin, serum albumin, serum uric acid, eGFR, cholesterol, triglyceride, high-density lipoprotein, low-density lipoprotein, and hs-CRP. *DM* diabetes mellitus, *CVD* cardiovascular disease, *BP* blood pressure, *eGFR* estimated glomerular filtration rate, *hs-CRP* high-sensitivity C-reactive protein, *OR* odds ratio, *CI* confidence interval

When adjusting for confounding factors, we found that elderly age, male sex, hypertension, hyperlipidemia, and lower levels of serum uric acid were associated with a high risk of coexisting DM and pre-existing CVD, DM, and pre-existing CVD versus the control. Of note, hypertension was strongly associated with a 13.72 (95% CI, 6.14 to 30.63)-fold risk of coexisting DM and pre-existing CVD versus the control. In addition, higher body mass index, systolic BP, hemoglobin, and cholesterol but lower diastolic BP were associated with a higher risk of the coexistence of DM and pre-existing CVD.

### Observational period and mortality

During 10,122.2 person-years of follow-up (median, 35.1 months; IQR, 17.9–61.7) months), 519 (17.7%) patients died, including 258 (43.4%) from CVD, 54 (10.4%) from infection, 9 (1.7%) from gastrointestinal bleeding, 16 (3.1%) from tumors, 93 (17.9%) from other causes, and 89 (17.1%) from unknown causes. In addition, 353 (12.0%) transferred to hemodialysis, 153 (5.2%) received renal transplants, 26 (0.9%) transferred to other dialysis centers, and 100 (3.4%) were lost to follow-up.

Table 3 shows that the incidence rates of all-cause deaths were 179.4, 102.4, 72.6, and 33.3/1000 patient-years, and the incidence rates of CVD deaths were 84.6, 49.4, 43.3, and 16.2/1000 patient-years among the DM plus CVD, DM, CVD, and control groups, respectively.

**Table 3:** The incidence rate of all-cause and CVD deaths^a^

	All-cause mortality	CVD mortality	Time at risk (years)	All-cause death incidence	CVD death incidence
Overall	519	258	10,122.2	51.3	25.5
Control group	256	125	7697.3	33.3	16.2
Pre-existing CVD group	57	34	785.6	72.6	43.3
DM group	117	57	1143.1	102.4	49.9
DM plus pre-existing CVD group	89	42	496.2	179.4	84.6

*DM* diabetes mellitus, *CVD* cardiovascular disease

^a^Incidence rate was calculated as number of events divided by total valid observational time at risk, scaled to episodes per 1000 years

**Fig. 1:**
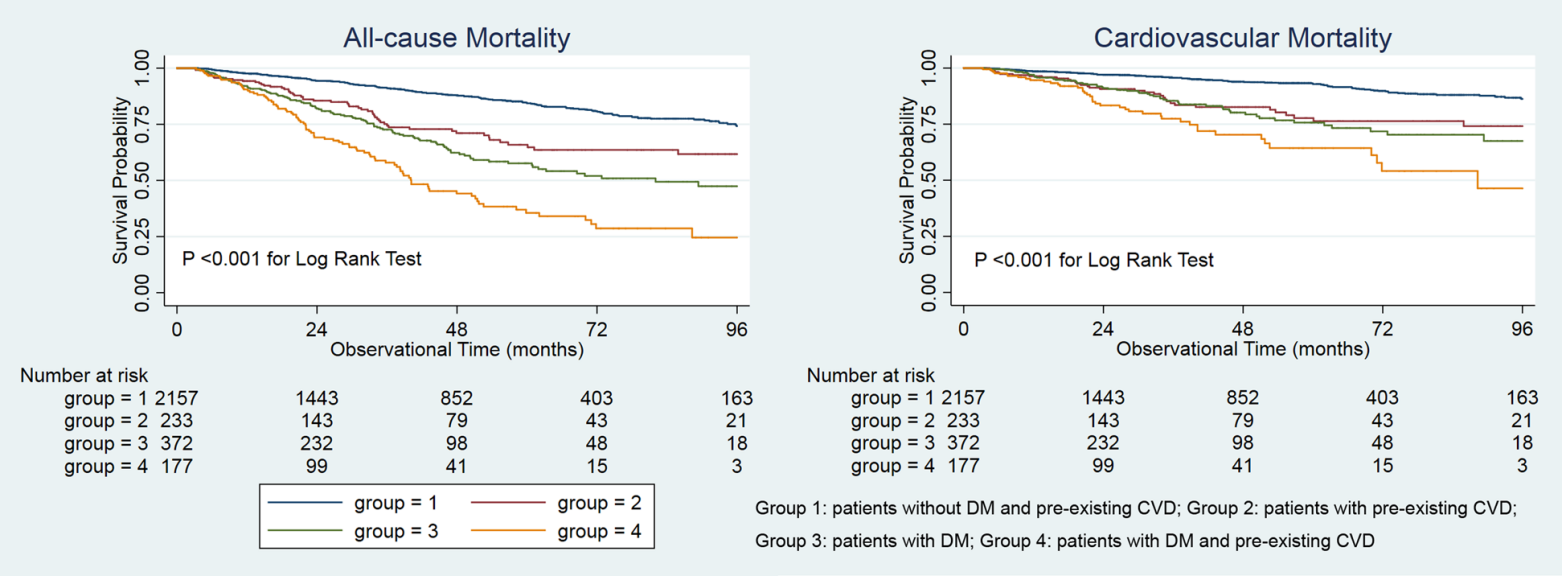
Survival probability for the four groups. DM, diabetes mellitus; CVD, cardiovascular disease

Survival analyses showed that patients with coexistent DM and pre-existing CVD had a poor survival probability compared with patients without DM and pre-existing CVD (Fig. 1).

The association between the coexistence of DM and pre-existing CVD and mortality was evaluated by different Cox proportional hazards regression models (Table 4).

**Table 4:** Associations between the coexistence of DM and CVD and mortality

	Model 1		Model 2		Model 3		Model 4	
	HR	95%CI	HR	95%CI	HR	95%CI	HR	95%CI
All-cause mortality								
Control group	Reference							
Pre-existing CVD group	2.22	1.66 to 2.95	1.41	1.05 to 1.89	1.41	1.05 to 1.89	1.43	1.07 to 1.92
DM group	3.13	2.51 to 3.89	1.87	1.48 to 2.35	1.87	1.48 to 2.35	1.89	1.50 to 2.38
DM plus pre-existing CVD group	5.56	4.36 to 7.08	2.78	2.13 to 3.63	2.78	2.13 to 3.63	2.85	2.18 to 3.72
*P* values for trend		< 0.001		< 0.001		< 0.001		< 0.001
CVD mortality								
Control group	Reference							
Pre-existing CVD group	2.70	1.85 to 3.94	1.82	1.23 to 2.69	1.82	1.23 to 2.69	1.82	1.23 to 2.68
DM group	3.13	2.29 to 4.28	1.93	1.39 to 2.69	1.93	1.39 to 2.69	1.88	1.35 to 2.61
DM plus pre-existing CVD group	5.39	3.80 to 7.66	2.87	1.95 to 4.22	2.87	1.95 to 4.22	2.79	1.91 to 4.08
*P* values for tend		< 0.001		< 0.001		< 0.001		< 0.001

Model 1, unadjusted; model 2, model 1 plus age, sex, body mass index, systolic BP, diastolic BP, current smoking, current alcohol consumption, 24-h urine volume, hypertension, and hyperlipidemia; model 3, model 2 plus medications; model 4, model 3 plus hemoglobin, serum albumin, serum uric acid, eGFR, cholesterol, triglyceride, high-density lipoprotein, low-density lipoprotein, and hs-CRP. *DM* diabetes mellitus, *CVD* cardiovascular disease, *BP* blood pressure, *eGFR* estimated glomerular filtration rate, *hs-CRP* high-sensitivity C-reactive protein, *HR* hazard ratio, *CI* confidence interval

Compared with no DM or pre-existing CVD, DM plus CVD, DM, and pre-existing CVD were associated with a 2.85 (95% CI 2.18 to 3.72), 1.89 (95% CI 1.50 to 2.38), and 1.43 (95% CI 1.07 to 1.92)-fold increased risk of all-cause mortality (P for trend < 0.001), and a 2.79 (95% CI 1.91 to 4.08), 1.88(95% CI 1.35 to 2.61), and 1.82 (95% CI 1.23 to 2.68)-fold increased risk for CVD mortality (P for trend < 0.001) in Model 4, respectively.

### Subgroup analyses

**Fig. 2:**
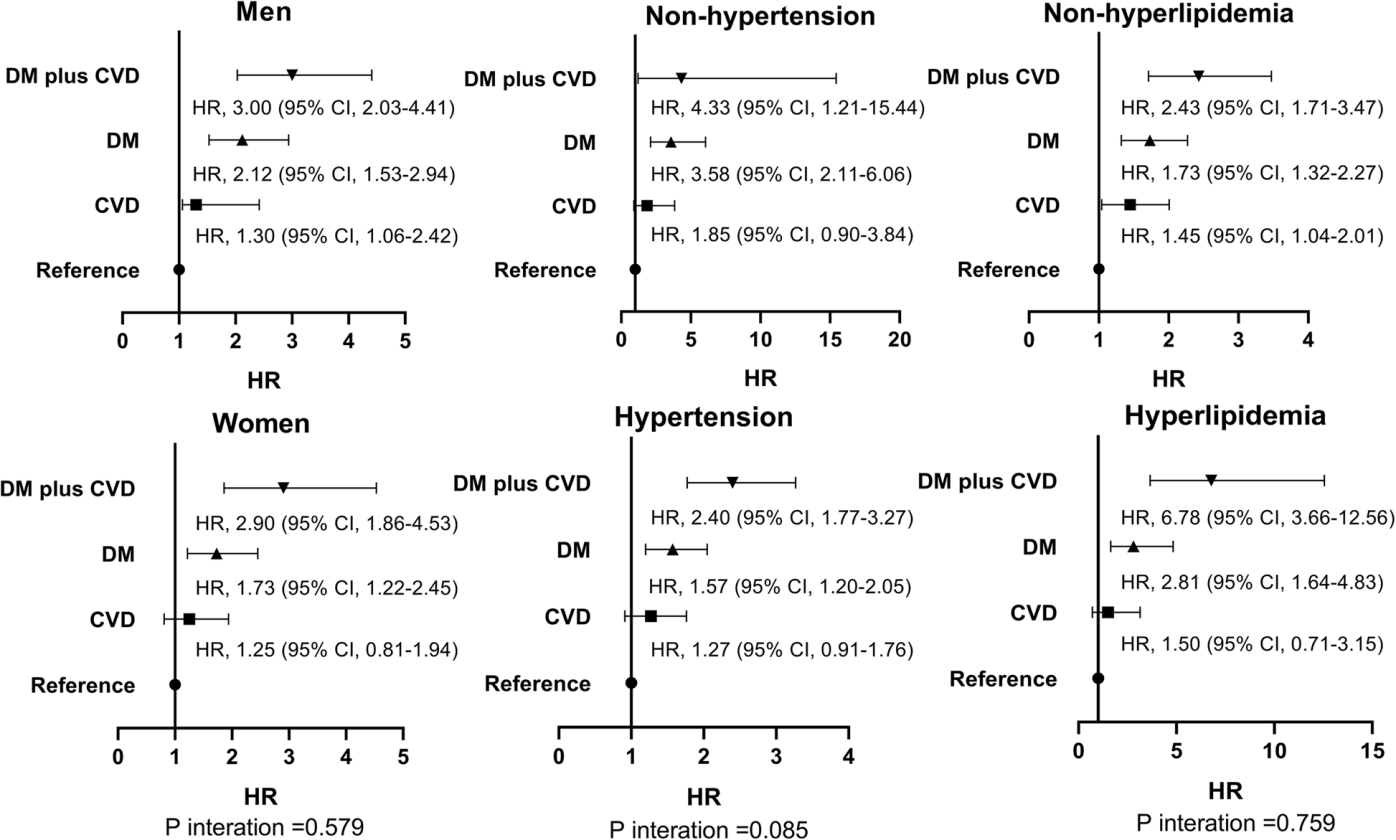
Association between the coexistence of DM plus pre-existing CVD and all-cause mortality among the subgroups. The factors in Model 4 were adjusted. DM, diabetes mellitus; CVD, cardiovascular disease; hazard ratio; CI, confidence interval

**Fig. 3:**
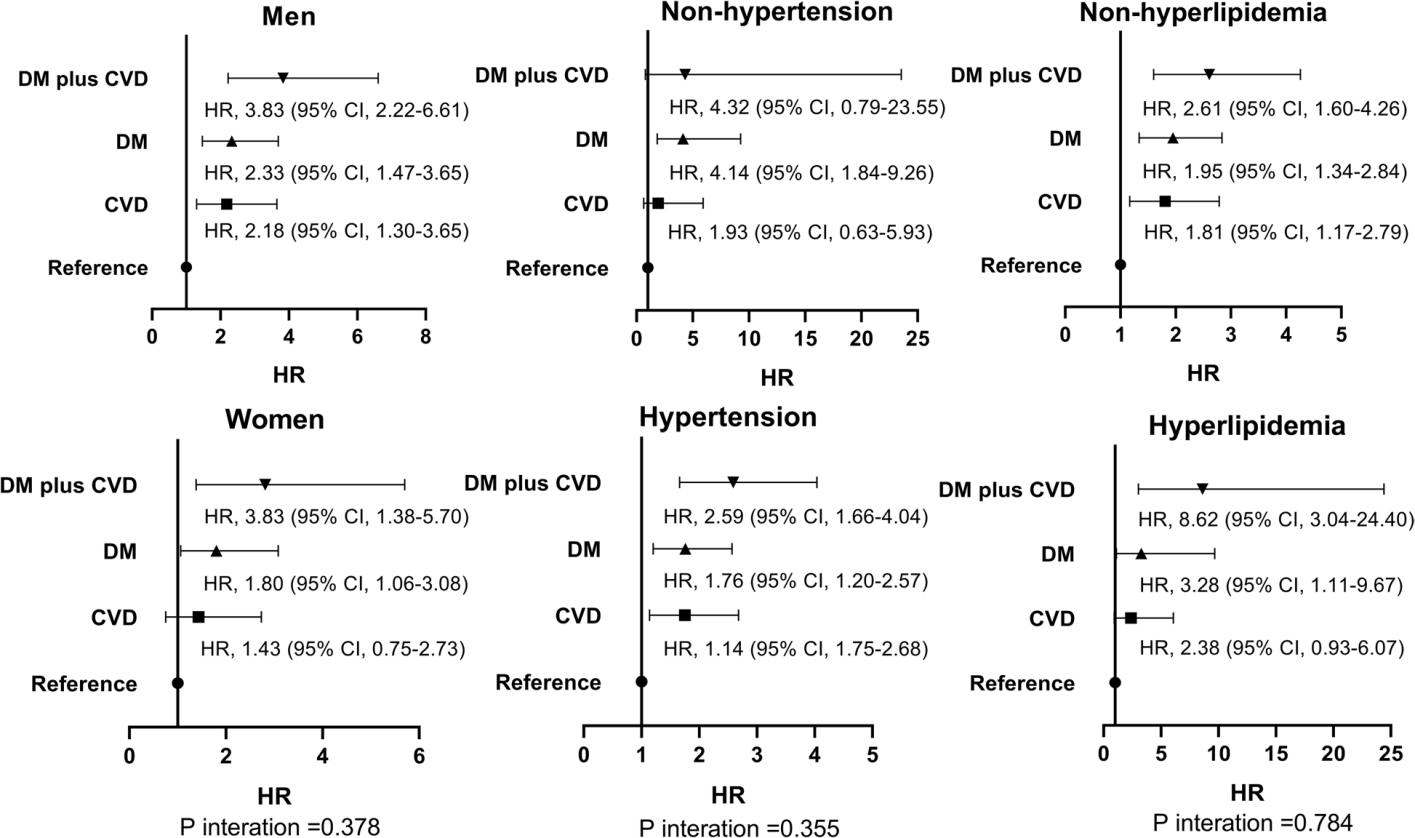
Association between the coexistence of DM plus pre-existing CVD and CVD mortality among the subgroups. The factors in Model 4 were adjusted. DM, diabetes mellitus; CVD, cardiovascular disease; hazard ratio; CI, confidence interval

Similar trends of the association between the coexistence of DM and pre-existing CVD and mortality were observed among subgroups of men, women, hypertension, nonhypertension, hyperlipidemia, and nonhyperlipidemia (Fig. 2 and Fig. 3).

*P* values for interactions were > 0.05 for all subgroups, suggesting that the increased risk of all-cause and CVD mortality associated with the coexistence of DM and pre-existing CVD was evident regardless of these variables. Table 5 shows the association between DM (pre-existing CVD as reference) and mortality. Compared with pre-existing CVD, DM was associated with a 1.46 (95% CI, 1.17 to 1.81)-fold higher risk of all-cause mortality and a 1.07 (95% CI, 0.69 to 1.67)-time higher risk of CVD mortality in Model 4.

**Table 5:** Associations between DM (pre-existing CVD as reference) and mortality^a^

	Model 1		Model 2		Model 3		Model 4	
	HR	95%CI	HR	95%CI	HR	95%CI	HR	95%CI
All-cause mortality								
Pre-existing CVD group	Reference							
DM group	1.49	1.20 to 1.86	1.41	1.10 to 1.80	1.37	1.07 to 1.75	1.46	1.17 to 1.81
CVD mortality								
Pre-existing CVD group	Reference							
DM group	1.13	0.74 to 1.73	1.04	0.67 to 1.62	1.06	0.68 to 1.65	1.07	0.69 to 1.67

^a^P interaction = 0.280 (all-cause mortality); P interaction = 0.113 (CVD mortality). Model 1, unadjusted; model 2, model 1 plus age, sex, body mass index, systolic BP, diastolic BP, current smoking, current alcohol consumption, 24-h urine volume, hypertension, and hyperlipidemia; model 3, model 2 plus medications; model 4, model 3 plus hemoglobin, serum albumin, serum uric acid, eGFR, cholesterol, triglyceride, high-density lipoprotein, low-density lipoprotein, and hs-CRP. *DM* diabetes mellitus, *CVD* cardiovascular disease, *BP* blood pressure, *eGFR* estimated glomerular filtration rate, *hs-CRP* high-sensitivity C-reactive protein, *HR* hazard ratio, *CI* confidence interval

There was no effect of an interaction between DM and pre-existing CVD on all-cause (P interaction = 0.280) or CVD mortality (P interaction = 0.113). Additionally, compared with pre-existing CVD, the coexistence of DM and pre-existing CVD was associated with a 2.07 (95% CI, 1.44 to 2.97)-fold higher risk of all-cause mortality and a 1.72 (95% CI, 1.05 to 2.82)-time higher risk of CVD mortality in Model 4. When compared with DM, the coexistence of DM and pre-existing CVD was associated with a 1.58 (95% CI, 1.18 to 2.10)-fold higher risk of all-cause mortality and a 1.60 (95% CI, 1.06 to 2.43)-time higher risk of CVD mortality in Model 4.

## Discussion

In our study, the coexistence of DM and pre-existing CVD at the start of dialysis was more strongly associated with a higher risk of all-cause and CVD mortality than either DM or pre-existing CVD alone. Similar trends were observed in subgroups of sex, hypertension status, and hyperlipidemia status. In addition, DM was associated with a higher risk of all-cause mortality but a similar risk of CVD mortality compared with pre-existing CVD.

In the general population, approximately one-third of patients with myocardial infarction have diabetes. The prognosis in terms of survival rates is worse for diabetic patients with coronary artery disease than for those with coronary artery disease but no diabetes [16, 17]. A previous study reported that DM patients with pre-existing CVD had a 3.3-fold higher risk of mortality than DM patients without pre-existing CVD [9]. In the dialysis population, pre-existing CVD was independently associated with a higher risk of mortality in dialysis patients [3, 18–20]. However, in dialysis patients, the association between the coexistence of DM and pre-existing CVD remains unknown. In our study, compared with patients without DM and pre-existing CVD, patients with coexistence of DM and pre-existing CVD, those with only DM, and those with only pre-existing CVD had a 2.85-, 1.89-, and 1.43-fold risk of all-cause mortality and a 2.79-, 1.88-, and 1.82-fold CVD mortality, respectively, suggesting that the coexistence of DM and pre-existing CVD was more strongly associated with the highest risk of all-cause and CVD mortality, followed by DM and pre-existing CVD accordingly. Patients with coexisting DM and pre-existing CVD at baseline were more likely to be older and have hypertension and hyperlipidemia. Older age and hypertension are associated with poor prognosis in dialysis [21–23]. Therefore, the coexistence of DM and pre-existing CVD represented poor health conditions in CAPD patients, contributing to a high risk of mortality. In the general population, diabetes mellitus is currently defined as a coronary artery disease equivalent [24]. However, we found that in CAPD patients, DM was associated with a higher risk of all-cause and CVD mortality than pre-existing CVD. In our research, we defined CVD as a complex of cardiac, cerebrovascular, and peripheral vascular diseases, not just coronary artery disease. Therefore, we comprehensively compared the effects of DM and existing pre-existing CVD on dialysis patient mortality.

The subgroup analyses' findings enhanced the consistency in the association between the coexistence of DM and pre-existing CVD and the risk of death from any cause and CVD across selected CVD risk factors. It was noteworthy that the strengths in the association between the coexistence of DM and pre-existing CVD and the risk of death from any cause and CVD may vary by sex, hypertension status, and hyperlipidemia status, with HRs ranging from 2.40 to 8.62 comparing the coexistence of DM and pre-existing CVD with no DM or pre-existing CVD. In hyperlipidemia patients, the coexistence of DM and pre-existing CVD was associated with an 8.62-fold higher risk of CVD mortality than the absence of DM and pre-existing CVD. However, in hypertension patients, the coexistence of DM and pre-existing CVD was associated with a 2.40-fold higher risk of all-cause mortality than the absence of DM and pre-existing CVD. There was no interaction between the coexistence of DM and pre-existing CVD and subgroups, suggesting that the increased risk of all-cause and CVD mortality associated with the coexistence of DM and pre-existing CVD was evident regardless of these variables. These subgroup analyses further supported the association of the coexistence of DM and pre-existing CVD and mortality. In addition, DM was associated with a 1.46-fold higher risk of all-cause mortality than pre-existing CVD and was at a similarly high risk of CVD mortality compared with pre-existing CVD. The findings suggested that DM was more strongly associated with non-CVD mortality than pre-existing CVD.

To date, there is no study regarding baseline factors associated with the coexistence of DM and pre-existing CVD. In our study, in CAPD patients, older age, male sex, higher body mass index, systolic BP, hemoglobin, hypertension, and hyperlipidemia, and lower diastolic BP and serum uric acid were independently associated with a high risk of the coexistence of DM and pre-existing CVD. It was noteworthy, however, that among the above factors, hypertension was the most strongly associated with the coexistence of DM and pre-existing CVD, followed by hyperlipidemia.

This study's strengths included a large sample size from five dialysis facilities, generalized inclusion, and a detailed assessment of and adjustment for all-cause and CVD risk factors. Several limitations should be considered. First, this was a retrospective study with potentially unaccounted for confounding factors and selection biases. However, after adjusting for confounding variables at baseline, we did not conclude the potential causal relationship between the coexistence of DM and pre-existing CVD and mortality. Second, we did not collect types of DM and DM-specified factors such as blood glucose, hypoglycemic medications, and DM duration, which may affect our findings. Third, the comorbidity diagnoses relied on the record data, and there may have been some disease misclassifications. Fourth, we did not take into account the severity of comorbidities. Last, although we only excluded those with age < 18 years or < 3-month follow-up, all eligible patients were from China, suggesting that our findings may lack generalization to other ethnic populations.

## Conclusions

In our study, in CAPD patients, the coexistence of DM and pre-existing CVD at the start of CAPD was associated with the highest risk of all-cause and CVD mortality, followed by DM and pre-existing CVD. In addition, compared to pre-existing CVD, DM was associated with a higher risk of all-cause mortality but a similar risk of CVD mortality. Our findings suggested that a combined assessment of the coexistence of DM and pre-existing CVD compared with a separate assessment of the two comorbidities further improved the risk stratification of CAPD patients at risk of mortality.

## Data Availability

The datasets used and/or analyzed during the current study are available from the corresponding author on reasonable request.
